# Determination of the Geographical Origin of Walnuts (*Juglans regia* L.) Using Near-Infrared Spectroscopy and Chemometrics ^†^

**DOI:** 10.3390/foods9121860

**Published:** 2020-12-13

**Authors:** Maike Arndt, Alissa Drees, Christian Ahlers, Markus Fischer

**Affiliations:** 1 Hamburg School of Food Science, Institute of Food Chemistry, University of Hamburg, Grindelallee 117, 20146 Hamburg, Germany; maike.arndt@chemie.uni-hamburg.de (M.A.); alissa.drees@chemie.uni-hamburg.de (A.D.); christian.ahlers@hotmail.de (C.A.); 2 Center for Hybrid Nanostructures (CHyN), Department of Physics, University of Hamburg, Luruper Chaussee 149, 22761 Hamburg, Germany

**Keywords:** walnut, FT-NIR, geographical origin, data pre-processing, *Juglans regia* L.

## Abstract

The prices of walnuts vary according to their geographical origin and, therefore, offer a financial incentive for adulteration. A reliable analysis method is required to quickly detect possible misdeclarations and thus prevent food fraud. In this study, a method to distinguish between seven geographical origins of walnuts using Fourier transform near-infrared (FT-NIR) spectroscopy combined with chemometrics as a fast, versatile, and easy to handle analytical tool was developed. NIR spectra of 212 ground and afterwards freeze-dried walnut samples, harvested in three consecutive years (2017–2019), were collected. We optimized the data pre-processing by applying and evaluating 50,545 different pre-processing combinations, followed by linear discriminant analysis (LDA) which was confirmed by nested cross-validation. The results show that in the scope of our research minimal pre-processing led to the best results: By applying just multiplicative scatter correction (MSC) and median centering, a classification accuracy of 77.00% ± 1.60% was achieved. Consequently, this complex model can be used to answer economically relevant questions e.g., to distinguish between European and Chinese walnuts. Furthermore, the great influence of the applied pre-processing methods, e.g., the selected wavenumber range, on the achieved classification accuracy is shown which underlines the importance of optimization of the pre-processing strategy.

## 1. Introduction

With over 2.5 million tons produced per year, *Juglans regia* L., the English or Persian walnut, is one of the most economically important nut crops [1]. Walnuts are widely consumed as a raw, toasted, or pickled snack, used as an ingredient in pastries, ice cream, muesli, and for the production of liquor or oil. Their broad acceptance occurs not only due to their special organoleptic characteristics, but also because of their high nutrition value that is associated with health benefits, such as the improvement of serum lipid profiles and a reduction of the risk for coronary heart disease [2,3,4]. Walnut trees grow in a mild climate without night frost during late spring or early fall [5,6,7]. Hence, the main producers are China, the USA, Iran, and Turkey. In 2018, China and California contributed, combined, about 60% of the worldwide walnut production [1]. Some walnuts from smaller harvesting areas such as France are, nevertheless, economically significant because of their particular sensory characteristics, which are closely linked to their geographical origin and are subject to special protection by labels such as “Protected Designation of Origin” (PDO) [8]. The different quality characteristics lead to significant price differences which can cause illegal mislabeling of the geographical origin of the product [9]. The misdeclaration of shelled Chinese walnuts as French would have, for example, in 2018 increased the profit by 30% [1,10]. In order to ensure the origin determination, the development of objective analytical methods is required. Currently, to the best of our knowledge, no effective, simple, and routinely applicable method for the origin determination of walnuts has been developed. While scientific studies have been made on the geographical discrimination through the fatty acid profile [11], tocopherol concentrations [12], and fingerprinting approaches with ^1^H-nuclear magnetic resonance (NMR) spectroscopy in combination with carbon-isotope ratio [13,14], none of these findings allow for an easily feasible and applicable analytical procedure. Gu et al. showed in a recent study the potential of near-infrared (NIR) spectroscopy to identify the geographical origin of walnuts within the region Xinjiang (China) [15].

NIR spectroscopy is a powerful tool to estimate quality attributes of various commodities and even of walnuts [16,17]. It features some main advantages: it is fast, easy to use, reliable, non-destructive, environmentally friendly since no hazardous chemicals are required, and hence suitable for on-line detection [18]. Due to its versatility, it is already established in industrial routine laboratories for many different food-related analyses, e.g., the analysis of oil processing parameters [19,20]. However, the accuracy of the analysis depends considerably on the applied chemometric treatment [21,22,23]. Many different spectra pre-processing strategies as well as a variety of classification methods have been used, depending on the matrices and research questions. Therefore, we evaluated the chemometric analysis in terms of the suitability for geographical origin determination of walnuts. Specifically, we conducted a “trial-and-error”—in combination with the “visual inspection”—approach according to Engel et al. [22], in which a variety of pre-processing combinations were applied followed by classification via linear discriminant analysis (LDA).

The aim of this study was to optimize the pre-processing strategy in order to develop a reliable near-infrared-based analytical approach for determining the geographical origin of walnuts suitable for routine use.

## 2. Materials and Methods

### 2.1. Walnut Sample Acquisition

A total of 212 walnut samples were acquired from seven different countries (Switzerland (CH), China (CN), France (FR), Italy (IT), Germany (DE), Hungary (HU), United States of America (USA)). For each sample, at least 100 g of dried walnuts (referring to shelled nuts, corresponding to about 250 g of dried walnuts in the shell) were purchased directly from producers or exporters to ensure the samples’ authenticity. Since walnut varieties can be distinguished by NIR spectroscopy [17], 41 different varieties were analyzed. This broad spectrum of varieties minimizes the correlation of varieties and geographical origin. The harvest year also has an impact on the NIR analysis [24,25,26]. Therefore, samples of three successive years (2017–2019) were acquired from each of the seven countries. The sample distribution can be found in Table 1. In addition, the detailed specification of all walnut samples including the varieties is given in Table S1.

### 2.2. Sample Preparation

The dried walnuts were cracked using a manual cracking machine (Wal Man Small, Feucht Obsttechnik GmbH, Erbstetten, Germany), if necessary. Subsequently, the shelled nuts were shock frozen in liquid nitrogen for at least 5 min to prevent alterations of the metabolome. Afterwards, the nuts were stored at −20 °C until further processing.

In a previous comparison of different sample preparation techniques, which were applied to almonds prior to NIR measurement, grounding combined with freeze-drying achieved the highest classification accuracies [27]. Due to the similarity of the matrices, the analyzed walnuts were ground and lyophilized for further analysis. Therefore, around 100 g of shelled walnuts were ground using a knife mill (Grindomix GM 300, Retsch, Haan, Germany). In order to prevent friction heat, the grounding was applied using dry ice in a ratio of 2/1 (*w/w* dry ice/walnuts). The ground walnut material was subsequently lyophilized for 48 h (Beta 1–8 LSCplus, Martin Christin Freeze Dryers GmbH, Osterode, Germany). After half of the lyophilization time (24 h), the sample material was manually stirred to ensure uniform freeze-drying.

### 2.3. Fourier Transform Near-Infrared (FT-NIR) Analysis

In order to represent the samples’ basic population, three technical replicates were measured. For this purpose, (1.25 ± 0.05) g frozen and lyophilized walnut ground material (in the following referred to as only ground material) were weighed three times in glass vials ((52.0 × 22.0 × 1.2) mm, Nipro Diagnostics Germany GmbH, Ratingen, Germany). The vials were then closed and stored for 0.5–3 h at room temperature (22 °C ± 2 °C) to thaw the ground material.

Each replicate was analyzed five times using a Fourier transform near-infrared (FT-NIR) spectrometer with an integration sphere (TANGO including the software OPUS 7.5 for data acquisition, Bruker Optics, Bremen, Germany). Between each data collection, the glass vial was shaken thoroughly. Before measurement, it was visually ensured that the filling level of the ground material was uniform. In result, this operation procedure lead to 15 spectra per sample. The spectra were acquired in a wavenumber range of 11,550–3950 cm^−1^ with 50 scans per spectrum and a resolution of 2 cm^−1^.

### 2.4. Spectra Pre-Processing

**Figure 1:**
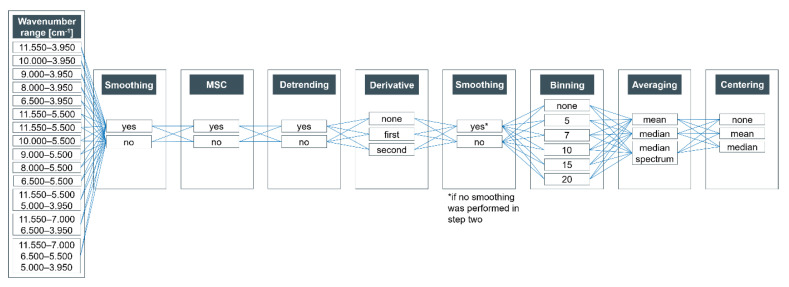
Flowchart showing all different combinations of pre-processing methods applied in the optimization. In total, all combinations lead to 50,545 different pre-processing strategies. Smoothing was performed either in second or sixth place, double smoothing in one pre-processing combination has not been performed; MSC—multiplicative scatter correction.

In order to optimize the pre-processing strategy for our analytical aim, the so called “Trial and error”—in combination with the “visual inspection”—approach according to Engel et al. [22] was performed. In the scope of this approach, various established pre-processing methods were applied. The processed data were then classified according to the geographical origin of these walnut samples (see Section 2.4.). Figure 1 shows the flowchart of all pre-processing steps. Following this scheme 50,545 different pre-processing combinations were obtained. These pre-processing combinations include every possibility and are not based on a manual selection. Therefore, it is reasonable that not every mathematical combination is suitable for our analytical aim. Hence, the verification of the plausibility of the strategies that achieved the highest classification accuracies is an important part of the optimization of the data pre-processing. The scheme (Figure 1) is based on seven different pre-processing methods:

1. Wavenumber reduction: In order to reduce e.g., noise, the wavenumber range used can be restricted. In addition, areas with unusable or result distorting signals—particularly water—are excludable and could affect the NIR screening results. Even if the samples are freeze-dried, small differences in water absorbance (bands at about 6900 cm^−1^ and 5155 cm^−1^ [28,29,30]) could have a major impact on the classification. The exclusion of the aforementioned wavenumber range was included in the shown optimization only based of the potentially negative influence of the water bands. Thus, the selection does not correspond to a classical variable selection approach. If more than one wavenumber range was used (e.g., 11,550–5500 cm^−1^ and 5000–3950 cm^−1^) the following pre-processing steps were applied separately for each wavenumber section to avoid artifacts. These individual sections were assembled together for classification after they had been fully processed [27,31,32].
2. Smoothing: Smoothing by a Savitzky–Golay filter can improve the signal-to-noise ratio (*S/N* ratio) and can be applied after the wavenumber reduction or later. If smoothing was applied at the second position of the pre-processing flow, an additional smoothing was omitted. In general, a second-order smoothing was performed with a frame size of three.
3. Multiplicative scatter correction: Multiplicative scatter correction (MSC) is a helpful and in many cases necessary pre-processing strategy to reduce additive and multiplicative effects caused e.g., by different particle sizes. If the data were corrected by MSC [33], the mean spectrum of all samples was used as a reference. Since all wavenumbers have an effect on the mean spectrum, MSC should be performed after wavenumber reduction.
4. Detrending: This (polynomial order = 2) was performed to reduce potential baseline shifts.
5. Derivative: Another pre-processing strategy for minoring baseline effects is the use of derivatives. The first derivative can reduce offsets and other additive effects, whereas baseline slopes and multiplicative effects can be diminished calculating the second derivative. In the scope of our research, we used gap-segment derivative with a window size of 11 and a filter length of 11.
6. Binning: This is an effective tool to reduce the computing time and noise. Since adjacent wavelengths are usually highly correlated in spectroscopic data, it is possible to average them depending on the window size without loss of information. As part of the optimization, binning windows from 1–20 (1, 5, 7, 10, 15, 20) were evaluated (see Figure 1).
7. Averaging: This procedure is an indispensable basis for classification. Only in this way reliable classification accuracies can be achieved. In addition to the arithmetic mean, the median can also be applied. The latter is more robust against outliers. An alternative, commonly used in NMR spectroscopy [34], is the selection of a median spectrum.
8. Centering: Various methods were applied: mean centering (mean = 0), median centering (median = 0) or none.

All pre-processing strategies and the resulting combinations were performed using Matlab 2019b (The MathWorks Inc., Natick, MA, USA).

### 2.5. Multivariate Data Analysis

LDA was applied to classify the walnut samples in regard to their geographical origin. LDA was selected to maximize inter-class variances while minimizing intra-class variances [35,36]. In order to obtain a robust model, nested cross-validation (cv) was performed [37,38]. Therefore, the data were split in five equal parts using stratified random sampling. Four parts formed the training set, and one part formed the test set. All subsets were mapped using a 5-fold outer cv. Additionally, the model parameters were optimized in a 10-fold inner cv. In the scope of LDA classification, the number of principal components (PCs) that the model is based on has to be optimized in the inner cv-loop. Since the sample splits have a major impact on the classification, the stratified random sampling has to be repeated. For identifying the less and the most suitable pre-processing combinations, a 5-fold repetition was performed (see Appendix A). In order to finally optimize the pre-processing strategy, the 100 combinations with the highest classification accuracies in 5-fold repetition were classified in a 20-fold repetition (see Appendix B).

For visualization and investigation purposed principal component analysis (PCA) based on the non-linear iterative partial least squares (NIPALS) algorithm was conducted as well [39,40]. The PCA was calculated based on the optimized pre-processing combination H7 (see Table 2). Strategy H7 includes MSC, smoothing at position 2, median centering and considers a wavenumber range of 8000–3950 cm^−1^. All mentioned multivariate data analyses were performed using Matlab 2019b (The MathWorks Inc., Natick, MA, USA). 

## 3. Results

### 3.1. Spectra Interpretation

**Figure 2:**
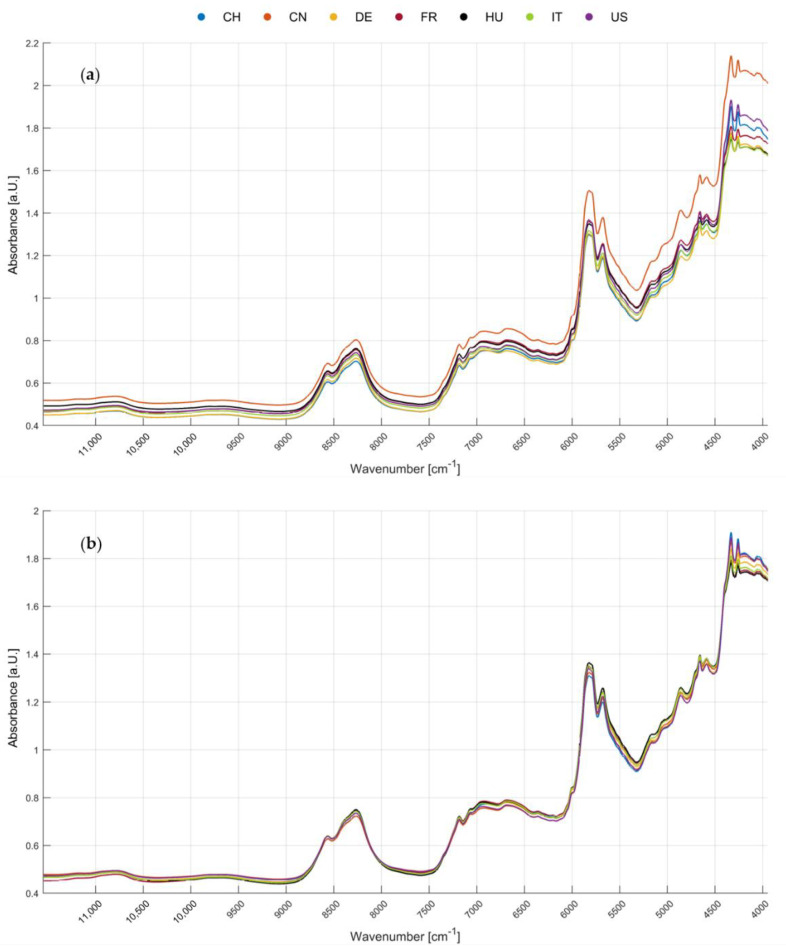
Mean spectra of the seven analyzed walnut origins in a wavenumber range of 11,550–3950 cm^−1^: (**a**) prior to further pre-processing; (**b**) MSC-corrected.

Figure 2 shows the median spectra of seven economically important walnut provenances (a) before and (b) after MSC in a wavenumber range of 11,550–3950 cm^−1^. It can be observed that in the wavenumber range from 11,550–9000 cm^−1^ only a few prominent bands appear, while the wavenumbers from 9000–3950 cm^−1^ contain a higher information density. Due to overlapping peaks caused by the complex matrix, a precise assignment of the peaks to specific metabolites or proteins is not possible. However, correlations between the bands and macronutrients can be assessed. As illustrated in Figure 2b, differences in peak intensity between the MSC-corrected mean spectra of the seven analyzed walnut origins are visible. By correlation of the bands which show absorbance differences to substance classes, predictions on potential substance classes for geographical origin determination can be made. For example, the band at 4900–4600 cm^−1^ is attributed to the N-H stretching and thereby linked to the protein content of the walnuts [28,41]. This wavenumber range has already been identified as relevant for the differentiation of the geographical origin of walnuts via NIR screening in another study [15].

The bands at approximately 5800 cm^−1^ and 4200 cm^−1^ exhibit intensity discrepancies. These bands are most likely caused by the first overtone of the C–H stretching of methylene and the C–H combination, respectively [28,41]. Hence, these bands can be associated with the lipid content of the examined material. Additionally, the band at around 4700 cm^−1^ is attributed to the C=C deformation and stretching of unsaturated lipids [28,41,42]. Therefore, lipids can be identified as a potential characteristic substance class for the determination of the geographical origin. Lipids are regulated by stress which is mainly caused by local exogenous factors [43,44]. Stress as a whole, therefore, represents a marker for geographical origin. The potential of lipids as biomarkers for the geographical origin of walnuts has been shown by Esteki et al., who used gas chromatographic fatty acid fingerprint analysis to classify walnuts from different geographical regions in Iran [11]. However, the fatty acid profile is also strongly dependent on the genotype of the walnuts, as has been shown by several groups [15,45,46,47]. In order to prevent bias by the variety on the geographical origin determination, 41 varieties of walnuts were included in the sample population.

**Figure 3:**
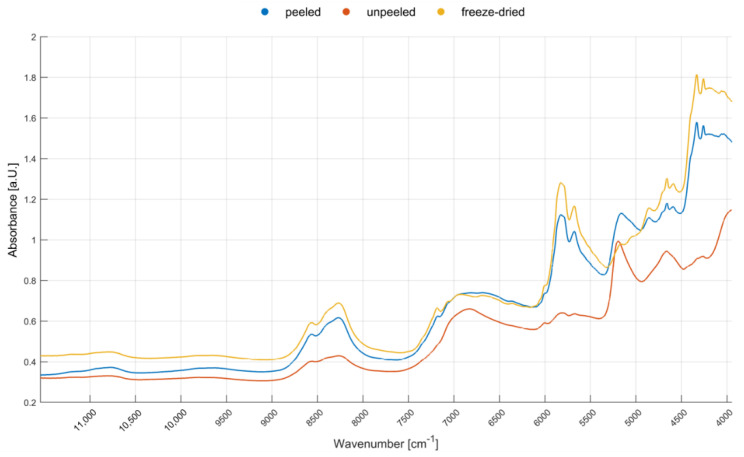
Mean spectra of an uncrushed peeled walnut, an uncrushed unpeeled walnut, and of a ground and freeze-dried walnut sample in a wavenumber range 11,550–3950 cm^−1^.

Figure 3 depicts the mean NIR spectrum of uncrushed walnuts (shelled) in comparison to those of ground and freeze-dried walnuts in a wavenumber range from 11,550–3950 cm^−1^. As shown, the three sample preparation techniques lead to significant differences in the spectra obtained. The unpeeled (with testa) whole walnuts’ spectrum overall features lower absorbance values, caused by a greater amount of light being reflected which may indicate specular surface reflection [48]. The mean spectrum of the peeled walnut (with manually removed testa) has more similar absorption values compared to the mean spectrum of lyophilized walnuts and could, therefore, be estimated to be a suitable sample preparation. However, walnuts are predominantly exported in shell or shelled (with testa) [10] and the removal of the testa is difficult depending on the variety and ripeness [49]. Furthermore, ground blanched almonds achieved lower classification accuracies than unpeeled ground almonds, indicating that the chemical composition of the almonds testa obviously correlates with the geographical origin [50]. This statement is presumably, due to the mostly analogous, high polyphenolic, chemical composition of almonds’ and walnuts’ testa [51,52] also applicable for walnuts. Additionally, the spectra of whole walnuts (with or without testa) exhibit broad absorption bands at approximately 7050–6600 cm^−1^ and 5300–5000 cm^−1^. These bands correspond with the water content and are caused by the first overtone O–H stretching and the O–H combination, respectively [28,29,30]. The water content depends mainly on the storage, the variety and post-harvest conditions and less on the geographical origin [32]. Furthermore, it cannot be ensured that the drying-conditions of the walnuts are absolutely identical before sampling. Moreover, the water-associated bands overlap especially with the carbohydrate-correlated bands, which are also caused by the aforementioned O–H stretching and combination [28,41]. Therefore, cutting of the water-associated bands—as has been carried out within some of the pre-processing strategies and which would be necessary for analysis of whole walnuts—results in information loss. The discrimination of the geographical origin is a very complex research question, and as such, the loss of information can significantly reduce the classification accuracy. We showed in a previous study that—in comparison to whole, bisected, and ground almonds—lyophilized almonds (after grinding) achieved the highest classification accuracy for geographical origin determination [27]. The differences in the walnut sample spectra of the three sample preparation techniques (see Figure 3) are strikingly similar to the differences obtained from the almond sample spectra of the mentioned study. Additionally, the trade of ground walnuts is accomplished as well, whole walnuts are not always available. Hence, the walnuts have been ground and the water content of the walnuts has afterwards been reduced and leveled out by freeze drying. Nonetheless, differences in the remaining water content may have an influence on the spectra [27].

### 3.2. Principal Component Analysis

**Figure 4:**
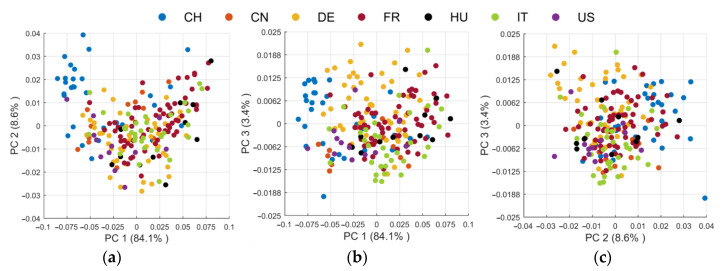
Principal component analysis (PCA) score plots of the 212 walnut samples after pre-processing combination H7 (see Table 5: (**a**) principal component (PC) 1 vs. PC 2; (**b**) PC 1 vs. PC 3; (**c**) PC 2 vs. PC 3.

In order to visualize the differences of the spectra, a PCA was conducted according to Section 2.4. Figure 4 shows the score plots of the seven analyzed walnut origins. In total, PC 1 and PC 2 represent 92.7% of the variance. However, as is observable in the plot depicted in Figure 4a, cluster trends of the geographical origins are not very pronounced along the first two principal components. For example, the Swiss walnuts are mainly spread at the upper left while the Hungarian walnuts are distributed on the right side of the PC 1 vs. PC 2 score plot. By adding PC 3, the variance explained is increased by 3.4 percentage points. A visual differentiation of the walnuts’ origin is not possible overall, although, based on Figure 4b,c, cluster trends are more predominant. For instance, the third principal component enhances the differentiation of German walnuts, which are spread at the top of the plots. Since the first three PCs do not cover the entire variance, Figure S1 shows the PCA score plots including PC 4. It is noticeable that even PC 1–PC 4 together are not sufficient for the differentiation of the geographical origin. Presumably, further variance which is caused by the geographical origin is represented by higher PCs. Therefore, we optimized the number of PCs in the inner cv-loop for the classification via LDA.

### 3.3. Optimization of Data Pre-Processing

The 50,545 pre-processing combinations achieved different classification accuracies in a range of 13.58–80.66% (5-fold repetition of nested cv, see Appendix A). First, this broad range of results underlines the importance of optimizing the data pre-processing. Within the results with the lowest classification accuracies (see Table 3), the influences can be quickly identified: higher binning windows and second derivative tend to lead to lower classification accuracies. A binning window of 20 leads to a significant reduction of information and is therefore not suitable for the pre-processing of our data. This is confirmed by the literature in which binning windows of up to ten are common [25,27,53,54]. Even if the use of the median spectra in combination with other pre-processing strategies leads to lower classification accuracies, this averaging method cannot be identified as the cause for low classification accuracies in general. Using other parameters in combination, a classification accuracy of up to 71% can be achieved using the median spectrum (see Appendix A). Furthermore, the averaging cannot be selected without an extensive inspection of the data set: If the data acquisition and sample material is sensitive to outliers, the median should always be used. In the scope of our research, for each sample 15 spectra were recorded to achieve a robust model. In addition, ground material was used ruling out an influence of the morphological differences. Therefore, the arithmetic mean can be used in our case. In order to provide a routine-applicable NIR screening, the number of multiple measurements can be reduced by using the median as averaging method. However, it has to be considered that this change may result in a lower classification accuracy (see Table 2).

Looking only at the pre-processing combinations that lead to the highest classification accuracies (classification accuracies based on LDA in a 20-fold repetition of the nested cv, see Appendix B, the selection was based on Appendix A), clear trends can be observed. The pre-processing techniques shown in Table 4 and Table 5 are composed of considerably fewer individual steps, showing that a multitude of data pre-processing is not always necessary to achieve reliable results. For example, combination H2 achieved a classification accuracy of 78.32% ± 2.19% without any pre-processing. These comparably high classification accuracies in relation to the small number of pre-processing strategies emphasize the low quantity of unwanted effects that need to be corrected. The fine grinding of the nuts ensured a uniform particle size (diameter of 100–500 µm), so the physical properties do not vary widely between the different groups. Nonetheless, considering Figure 2a, it has to be stated that additive and multiplicative scattering effects are still apparent in the spectra. In particular, the mean spectrum of the Chinese walnut samples is vertically shifted caused by a markedly different texture of the samples. Therefore, the scattering effects should be reduced in the context of data pre-processing to ensure comparability of the samples. As mentioned in Section 2.3., offsets can be minimized by MSC, detrending or derivatives. Hence, the most effective pre-processing combination including an offset-correction—here MSC—for determining the geographical origin of walnuts is combination H7 reaching a classification accuracy of 77.00% ± 1.60%. Thus, the most suitable data pre-processing strategy cannot be selected solely on the basis of classification accuracies and an additional inspection of the spectra as second step of optimization is indispensable.

For following pre-processing optimizations, it is shown that the use of different centering methods has less influence on the classification accuracy and thus plays a subordinate role. Since the data pre-processing combinations shown in Table 4 and Table 5 are based on reduced wavenumber ranges, the selection of the wavelength range is even more important. In many studies, the whole analyzed range (around 11,550–3950 cm^−1^) is used, because elimination of wavenumbers could lead to a loss of information [21,55,56]. In the aforementioned spectra (see Figure 2), a lower information density is apparent in the range >9000 cm^−1^, which rather deteriorates than improves the classification accuracy. Therefore, including the entire wavenumber range leads to a remarkable reduction of the model’s performance: Table 2 shows that the classification accuracy is reduced by about five percentage points if only the wavenumber range in strategy H7 is changed.

It can be concluded that an optimization of the data pre-processing is essential but no transferability to other matrices or research aims can be guaranteed. For example, other research groups, which are also working on NIR-based methods for detecting the origin of nuts, use different pre-processing strategies, although the matrix is in some cases very similar or identical [15,50,55,56]. For further, even non-targeted, approaches more emphasis should be put to the selection of the wavenumber range since this has a remarkable effect on the classification.

### 3.4. Classification of the Geographical Origin

**Figure 5:**
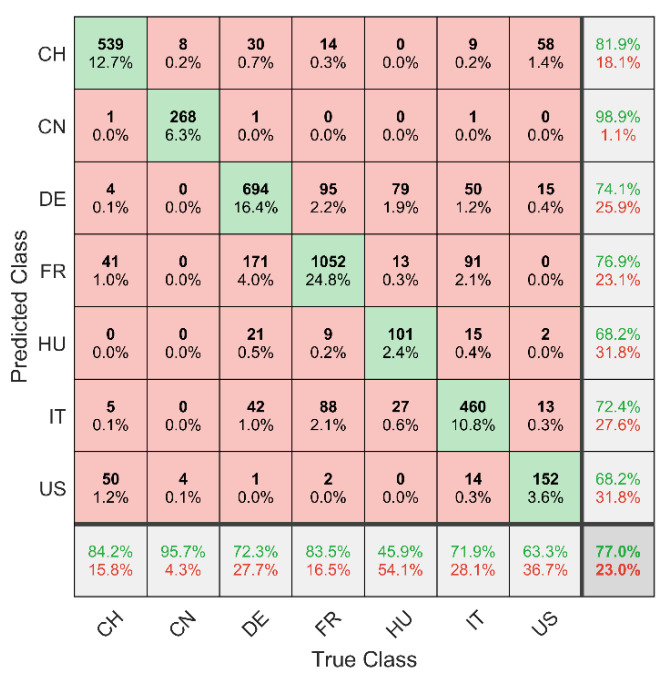
Confusion matrix based on all 212 walnut samples and using the optimized data pre-processing combination (see Table 5, strategy H7). The LDA leads to an overall classification accuracy of 77.00% ± 1.60% (see lower right corner). The values (given in counts) were summed over the 20 repetitions of the nested cv.

Using LDA as classifier, the optimized data pre-processing (strategy H7, see Table 5) leads to an overall classification accuracy *a* of 77.00% ± 1.60%. The corresponding confusion matrix is presented in Figure 5. The “true class” of the samples is given by the columns, while the rows show the predicted class affiliation by the multivariate classification model (here LDA). Hence, the diagonal shows all correctly classified walnut samples (green fields). The numbers depict the sum of the 20-fold cross-validation of the sample classification (see Section 2.4.) and are given in counts and in percentages. Furthermore, the resulting classification accuracies for the respective countries and the overall accuracy (see lower right corner) are given. The aforementioned performance represents a relatively high accuracy considering the model’s complexity. According to Segelke et al., the evaluation of the performance of a classification model also has to include the number of different classes *c* [57]. First, the random distribution *r* is calculated according to Equation (1). This parameter represents the probability to achieve a correct classification randomly and thus incorporate the number of different classes. Considering the random distribution (see Equation (2)) of our seven-class model, the accuracy-to-random ratio *a/r* would be 5.35 (see Equation (3)). An exemplary two-class model (*r* = 50%) with a classification accuracy of 90% would correspond to an accuracy-to-random ratio of 1.8 and hence to a less powerful model [57].

(1) $$r\;=\;\frac{100\%}{c}$$

(2) $$r\;\left(\mathrm{present}\;\mathrm{model}\right)\;=\;\frac{100\%}{7}\;=\;14.29\%$$

(3) $$\frac{a}{r}\;\left(\mathrm{present}\;\mathrm{model}\right)\;=\;\frac{77.00\%}{14.29\%}\;=\;5.35$$

With the multiclass model developed in this study, economically relevant questions can be addressed comprehensively: (i) Chinese walnuts are currently traded at relatively low prices [1,10], but can be distinguished from all other walnut origins with an accuracy of 95.7%. (ii) Likewise, the more expensive French walnuts can be distinguished from Chinese, Hungarian and US-American nuts with an accuracy of 99.13% (11 out of 1260 runs) [1,10]. Due to its geographical proximity, 7.5% (95 out of 1260 runs) of French walnuts are identified as German. To support the NIR screening, the morphology can be consulted here, since the classic varieties from France differ slightly in shape from the walnuts grown in Germany [58]. It must be explicitly stated that the walnuts’ morphology is also influenced by other factors such as the position on the tree [59]. However, some varieties are also cultivated in France and Germany, which makes a sensory analysis difficult.

## 4. Conclusions

In conclusion, the present study clearly shows that NIR spectroscopy is suitable for determining the geographical origin of walnuts. In order to obtain the most suitable pre-processing strategy in the course of this study, 50,545 pre-processing strategies were applied, such as MSC, detrending, derivations, smoothing, and combinations in various ways. In the scope of our research, minimal pre-processing led to the best results: using just MSC and median centering, a classification accuracy of 77.00% ± 1.60% was achieved via LDA. In addition, the selection of the wavenumber range has a major impact in the optimization. More variables—and thus more information—does not necessarily lead to better predictions i.e., the aforementioned accuracy can be achieved even with a reduced wavenumber range of 8000–3950 cm^−1^.

The NIR screening method developed includes seven different geographical origins of walnuts. Due to the simplicity of the data acquisition and the low cost, NIR screening can be easily transferred to quality assurance laboratories of small or medium-sized companies. This approach can be used in incoming goods inspection to verify the authenticity of a food’s raw material. In the context of this application, downscaling to a portable NIR spectrometer may be useful, although the transferability to such a device has yet to be tested.

In the long term, our model should be expanded to include other producing countries, such as Chile—a country that also cultivates and exports large quantities of walnuts. The data set should also be continuously supplemented with new harvest years to ensure the robustness and timeliness of the screening. It is also feasible to extend the NIR data by data fusion in order to obtain a more detailed fingerprint of the samples [60]. Therefore, it is crucial to use orthogonal methods e.g., mass spectrometry (MS) in order to complement the acquired information in the best way possible [61].

## Figures and Tables

**Table 1:** Samples distribution of all 212 walnut samples analyzed. A detailed listing of all samples is shown in Table S1.

Country of Origin	Samples	Major Varieties
Switzerland	31	Various
China	13	Chandler, Tulare
France	63	Fernor, Franquette, Lara
Italy	33	Chandler, Lara, Tulare
Germany	49	Various
Hungary	11	Various
USA	12	Various

**Table 2:** Pre-processing strategies based on combination H7—showing the impact of changing one parameter at a time (highlighted as **bold**) on the classification accuracy. The aforementioned classification accuracies are based on LDA of all 212 walnut samples in a 20-fold repetition of the nested cv.

	H7	H7-1	H7-2	H7-3	H7-4
Wavenumber range [cm^−1^]	8000–3950	**11,000–3950**	8000–3950	8000–3950	8000–3950
Smoothing	no	no	no	no	no
MSC	yes	yes	yes	yes	yes
Detrending	no	no	no	no	no
Derivative	none	none	**first**	**second**	none
Binning	1	1	1	1	1
Averaging	mean	mean	mean	mean	**median**
Centering	median	median	median	median	median
Classification Accuracy [%]	77.00	72.70	61.60	49.10	61.00
Standard Deviation [%]	1.60	1.70	2.00	2.90	2.34

cv: cross-validation, LDA: linear discriminant analysis, H7: combination with the seventh highest classification accuracy of the 50,454 strategies.

**Table 3:** Less suitable pre-processing strategies of all 50,545 combinations, based on linear discriminant analysis (LDA) in a 5-fold repetition of the nested cross-validation (cv) including 212 walnut samples.

	L1	L2	L3	L4	L5
Wavenumber range [cm^−1^]	11,550–3950				11,550–6500
Smoothing	no	no	position 2	position 6	position 6
MSC	yes	yes	yes	no	yes
Detrending	no	no	no	yes	no
Derivative	second	second	second	none	second
Binning	20	20	20	20	7
Averaging	median spectrum				median
Centering	mean	none	median	none	mean
Classification Accuracy [%]	13.58	13.77	13.87	14.25	14.43
Standard Deviation [%]	2.34	2.93	2.55	1.61	2.13

cv: cross-validation, LDA: linear discriminant analysis, L1–L5: combinations with the lowest (L) classification accuracies of the 50,454 strategies.

**Table 4:** Pre-processing strategies with the highest classification accuracies of all 50,545 combinations—part 1. The selection is based on LDA in a 5-fold repetition of the nested cv. The shown classification accuracies are based on all 212 walnut samples and a 20-fold repetition of the cv.

	H1	H2	H3	H4	H5
Wavenumber range [cm^−1^]	8000–3950				
Smoothing	position 2	no	position 2	No	position 2
MSC	no	no	no	No	no
Detrending	no	no	no	No	no
Derivative	none	none	none	None	none
Binning	1	1	1	1	1
Averaging	mean	mean	mean	Mean	mean
Centering	median	none	none	Median	mean
Classification accuracy [%]	79.02	78.32	77.99	77.87	77.48
Standard deviation [%]	1.61	2.19	2.37	1.95	2.32

cv: cross-validation, LDA: linear discriminant analysis, H1–H5: combinations with the highest (H) classification accuracies of the 50,454 strategies.

**Table 5:** Pre-processing strategies with the highest classification accuracies of all 50,545 combinations—part 2. The selection based on LDA in a 5-fold repetition of the nested cv. The classification accuracies shown are based on all 212 walnut samples and a 20-fold repetition of the cv. The highlighted (**bold**) pre-processing combination H7 has been chosen for further determination of the geographical origin of walnuts.

	H6	H7	H8	H9	H10
Wavenumber range [cm^−1^]	8000–3950	**8000–3950**	9000–3950	8000–3950	8000–3950
Smoothing	no	**no**	no	no	position 2
MSC	no	**yes**	no	yes	no
Detrending	no	**no**	no	no	no
Derivative	none	**none**	none	none	none
Binning	1	**1**	1	1	1
Averaging	mean	**mean**	mean	mean	mean
Centering	mean	**median**	none	mean	median
Classification accuracy [%]	77.08	**77.00**	76.70	76.44	76.39
Standard deviation [%]	1.68	**1.60**	2.17	1.57	2.15

cv: cross-validation, LDA: linear discriminant analysis, H**6**–H**10**: combinations with the highest (H) classification accuracies of the 50,454 strategies.

## Supplementary Materials

The following are available online at https://www.mdpi.com/2304-8158/9/12/1860/s1, Table S1: Overview of all walnut samples with country of origin, harvest year and variety; Figure S1: PCA score plots of the 212 walnut samples after pre-processing combination H7 (see Table 5): (a) PC 1 vs. PC 4; (b) PC 2 vs. PC 4; (c) PC 3 vs. PC 4; Figure S2: First derivative of MSC-corrected mean spectra of the seven analyzed walnut origins in a wavenumber range of 11,550–3950 cm^−1^.

## Appendix A

Classification accuracies of all 50,545 data pre-processing combination based on 212 walnuts samples of seven countries. The values mentioned refer to a classification via linear discriminant analysis in a 5-fold repetition of nested cross-validation.

## Appendix B

Classification accuracies of the 100 data pre-processing combinations achieving the highest results in 5-fold nested cross-validation (see Appendix A). The values mentioned refer to a classification via linear discriminant analysis in a 20-fold repetition of nested cross-validation.
