# Supplementary material for: Determination of the Geographical Origin of Walnuts (Juglans regia L.) Using Near-Infrared Spectroscopy and Chemometrics †
Source: Foods. 2020 Dec 13;9(12):1860. doi: 10.3390/foods9121860 (PMC7764259; doi:10.3390/foods9121860)
Supplement: Supplementary file 1 [file foods-09-01860-s001.pdf]

foods

Supplementary Materials

Determination of the Geographical Origin of Walnuts (*Juglans regia* L.) Using Near-Infrared Spectroscopy and Chemometrics †

Maike Arndt [1,‡], Alissa Drees [1,‡], Christian Ahlers [1] and Markus Fischer [1,2,*]

[1] Hamburg School of Food Science, Institute of Food Chemistry, University of Hamburg, Grindelallee 117, 20146 Hamburg, Germany; maike.arndt@chemie.uni-hamburg.de (M.A.); alissa.drees@chemie.uni-hamburg.de (A.D.); christian.ahlers@hotmail.de (C.A.)
[2] Center for Hybrid Nanostructures (CHyN), Department of Physics, University of Hamburg, Luruper Chaussee 149, 22761 Hamburg, Germany
* Correspondence: markus.fischer@uni-hamburg.de
† This article is dedicated to Prof. Francois Diederich on the occasion of his death in autumn 2020.
‡ Authors contributed equally to this work.

Received: 31 October 2020; Accepted: 11 December 2020; Published: 13 December 2020

Table 1. Overview of all walnut samples with country of origin, harvest year and variety.

Sample ID	Harvest year	Country of origin	Variety
17-CH-004	2017	Switzerland	Geisenheimer No. 26
18-CH-006	2018	Switzerland	Geisenheimer No. 26
18-CH-007	2018	Switzerland	Hartley
18-CH-008	2018	Switzerland	Fernette
18-CH-009	2018	Switzerland	Pedro
18-CH-010	2018	Switzerland	Serr
18-CH-011	2018	Switzerland	Broadview
18-CH-012	2018	Switzerland	Vina
18-CH-013	2018	Switzerland	Lara
18-CH-014	2018	Switzerland	Franquette
18-CH-015	2018	Switzerland	Chandler
18-CH-016	2018	Switzerland	Fernor
18-CH-017	2018	Switzerland	Parisienne
19-CH-018	2019	Switzerland	Chandler
19-CH-019	2019	Switzerland	Fernor
19-CH-020	2019	Switzerland	Meylannaise
19-CH-024	2019	Switzerland	Plovdivski
19-CH-025	2019	Switzerland	Milotai kensei
19-CH-026	2019	Switzerland	Ferbel
19-CH-027	2019	Switzerland	Ferjean
19-CH-028	2019	Switzerland	Lara
19-CH-029	2019	Switzerland	Feradan
19-CH-030	2019	Switzerland	Feroutte
19-CH-031	2019	Switzerland	Franquette
19-CH-032	2019	Switzerland	Milotai bötermö
19-CH-033	2019	Switzerland	Milotai 10
19-CH-034	2019	Switzerland	Saturn
19-CH-035	2019	Switzerland	Mars
19-CH-036	2019	Switzerland	Geisenheimer No. 139
19-CH-037	2019	Switzerland	Broadview

19-CH-038	2019	Switzerland	Dryanovski
**Sample ID**	**Harvest year**	**Country of origin**	**Variety**
17-CN-001	2017	China	NA
17-CN-002	2017	China	NA
17-CN-005	2017	China	Tulare
18-CN-007	2018	China	NA
18-CN-009	2018	China	Chandler
18-CN-010	2018	China	NA
18-CN-011	2018	China	NA
18-CN-013	2018	China	NA
19-CN-014	2019	China	Tulare
19-CN-015	2019	China	NA
19-CN-016	2019	China	Chandler
19-CN-017	2019	China	NA
19-CN-018	2019	China	NA
17-DE-005	2017	Germany	NA
17-DE-006	2017	Germany	NA
17-DE-007	2017	Germany	NA
17-DE-008	2017	Germany	NA
17-DE-010	2017	Germany	NA
18-DE-012	2018	Germany	NA
18-DE-013	2018	Germany	NA
18-DE-014	2018	Germany	NA
18-DE-015	2018	Germany	Lara
18-DE-016	2018	Germany	Weinsberg 1
18-DE-017	2018	Germany	Mars
18-DE-018	2018	Germany	Seifersdorfer Runde
18-DE-019	2018	Germany	Weidenheimer No. 139
18-DE-020	2018	Germany	Hartley
18-DE-021	2018	Germany	Franquette
18-DE-022	2018	Germany	Geisenheimer No. 139
18-DE-023	2018	Germany	Geisenheimer No. 286
18-DE-024	2018	Germany	Geisenheimer No. 120
18-DE-025	2018	Germany	Geisenheimer No. 1247
18-DE-026	2018	Germany	Geisenheimer No. 26
18-DE-027	2018	Germany	Esterhazy II
18-DE-028	2018	Germany	Geisenheimer No. 1239
18-DE-029	2018	Germany	Weinsberg 1
18-DE-030	2018	Germany	NA
18-DE-031	2018	Germany	NA
18-DE-032	2018	Germany	NA
19-DE-033	2019	Germany	NA
19-DE-034	2019	Germany	NA
19-DE-035	2019	Germany	NA
19-DE-036	2019	Germany	NA
19-DE-037	2019	Germany	Esterhazy II
19-DE-038	2019	Germany	Geisenheimer No. 139
19-DE-039	2019	Germany	Geisenheimer No. 286
19-DE-040	2019	Germany	Geisenheimer No. 120
19-DE-041	2019	Germany	Geisenheimer No. 1239
19-DE-042	2019	Germany	Geisenheimer No. 26
19-DE-043	2019	Germany	NA
19-DE-045	2019	Germany	Geisenheimer No. 26
**Sample ID**	**Harvest year**	**Country of origin**	**Variety**
19-DE-046	2019	Germany	Geisenheimer No. 138
19-DE-047	2019	Germany	Martlog

19-DE-048	2019	Germany	Milotai 10
19-DE-049	2019	Germany	Esterhazy II
19-DE-050	2019	Germany	Mars
19-DE-051	2019	Germany	Lara
19-DE-052	2019	Germany	NA
19-DE-053	2019	Germany	NA
19-DE-054	2019	Germany	NA
19-DE-055	2019	Germany	NA
19-DE-056	2019	Germany	NA
17-FR-003	2017	France	NA
17-FR-004	2017	France	Lara
17-FR-008	2017	France	Franquette
17-FR-009	2017	France	Lara
17-FR-010	2017	France	Fernor
17-FR-011	2017	France	Lara
17-FR-012	2017	France	Franquette
17-FR-013	2017	France	Franquette
17-FR-014	2017	France	Lara
17-FR-015	2017	France	Fernor
17-FR-016	2017	France	Lara
17-FR-017	2017	France	Lara
17-FR-018	2017	France	Lara
17-FR-019	2017	France	Franquette
17-FR-020	2017	France	Lara
17-FR-021	2017	France	Lara
17-FR-022	2017	France	Franquette
17-FR-023	2017	France	Lara
17-FR-024	2017	France	Fernor
17-FR-025	2017	France	Franquette
17-FR-026	2017	France	Lara
17-FR-027	2017	France	Fernor
18-FR-028	2018	France	NA
18-FR-030	2018	France	Lara
18-FR-031	2018	France	Lara
18-FR-032	2018	France	Lara
18-FR-033	2018	France	Lara
18-FR-034	2018	France	Lara
18-FR-035	2018	France	Lara
18-FR-036	2018	France	Lara
18-FR-037	2018	France	Fernor
18-FR-038	2018	France	Franquette
18-FR-039	2018	France	Franquette
18-FR-040	2018	France	Franquette
18-FR-041	2018	France	Franquette
18-FR-043	2018	France	Franquette
19-FR-044	2019	France	NA
19-FR-045	2019	France	Lara
19-FR-046	2019	France	Lara
19-FR-047	2019	France	Lara
**Sample ID**	**Harvest year**	**Country of origin**	**Variety**
19-FR-048	2019	France	Lara
19-FR-049	2019	France	Lara
19-FR-050	2019	France	Lara
19-FR-051	2019	France	Franquette
19-FR-052	2019	France	Franquette
19-FR-053	2019	France	Franquette

19-FR-054	2019	France	Lara
19-FR-055	2019	France	Franquette
19-FR-056	2019	France	Fernor
19-FR-057	2019	France	Lara
19-FR-058	2019	France	Lara
19-FR-059	2019	France	Fernor
19-FR-060	2019	France	Franquette
19-FR-061	2019	France	Fernor
19-FR-062	2019	France	Lara
19-FR-063	2019	France	Franquette
19-FR-064	2019	France	Lara
19-FR-065	2019	France	Lara
19-FR-066	2019	France	NA
19-FR-067	2019	France	Franquette
19-FR-068	2019	France	Franquette
19-FR-069	2019	France	NA
19-FR-070	2019	France	NA
17-HU-001	2017	Hungary	Milotai Botermo
17-HU-002	2017	Hungary	Milotai 10
17-HU-003	2017	Hungary	A 117
17-HU-004	2017	Hungary	NA
17-HU-005	2017	Hungary	NA
17-HU-006	2017	Hungary	NA
18-HU-007	2018	Hungary	NA
18-HU-009	2018	Hungary	A 117
18-HU-010	2018	Hungary	NA
18-HU-011	2018	Hungary	NA
19-HU-012	2019	Hungary	NA
17-IT-001	2017	Italy	Chandler
17-IT-002	2017	Italy	Tulare
17-IT-003	2017	Italy	Lara
18-IT-004	2018	Italy	Tulare
18-IT-005	2018	Italy	Chandler
18-IT-006	2018	Italy	Lara
18-IT-007	2018	Italy	Tulare
18-IT-008	2018	Italy	Chandler
18-IT-009	2018	Italy	Lara
18-IT-010	2018	Italy	Tulare
18-IT-011	2018	Italy	Chandler
18-IT-012	2018	Italy	Lara
18-IT-013	2018	Italy	Tulare
18-IT-014	2018	Italy	Chandler
18-IT-015	2018	Italy	Lara
18-IT-016	2018	Italy	Tulare
18-IT-017	2018	Italy	Chandler
**Sample ID**	**Harvest year**	**Country of origin**	**Variety**
18-IT-018	2018	Italy	Lara
19-IT-019	2019	Italy	Tulare
19-IT-020	2019	Italy	Chandler
19-IT-021	2019	Italy	Lara
19-IT-022	2019	Italy	Tulare
19-IT-023	2019	Italy	Chandler
19-IT-024	2019	Italy	Lara
19-IT-025	2019	Italy	Lara
19-IT-026	2019	Italy	Chandler
19-IT-027	2019	Italy	Lara

19-IT-028	2019	Italy	Tulare
19-IT-029	2019	Italy	Chandler
19-IT-030	2019	Italy	Lara
19-IT-031	2019	Italy	Lara
19-IT-032	2019	Italy	Chandler
19-IT-033	2019	Italy	Lara
17-US-007	2017	USA	Chandler
18-US-009	2018	USA	NA
18-US-010	2018	USA	Tulare
18-US-011	2018	USA	Livermore
18-US-012	2018	USA	NA
18-US-013	2018	USA	NA
18-US-014	2018	USA	Serr
18-US-015	2018	USA	Chandler
19-US-017	2019	USA	Chandler
19-US-018	2019	USA	Franquette
19-US-019	2019	USA	Tulare
19-US-020	2019	USA	Howard

NA: not available, USA: United States of America.

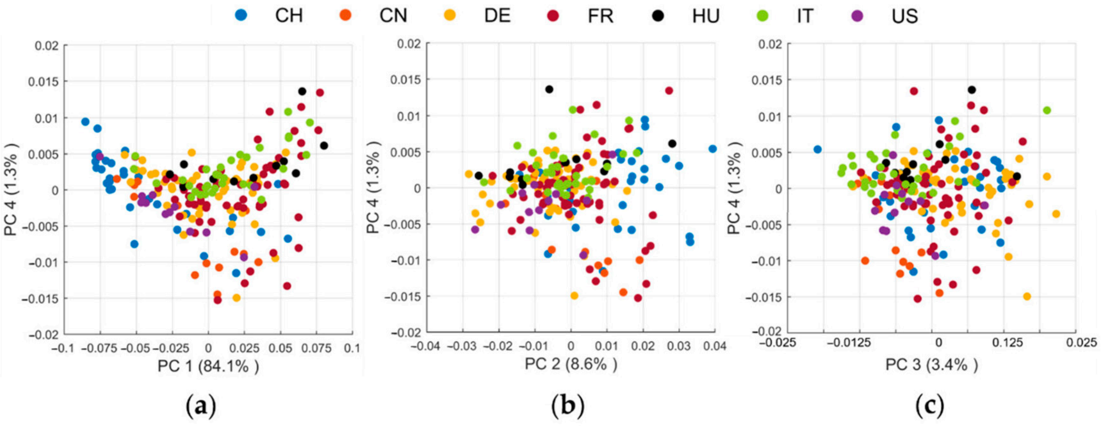

Figure 1. PCA score plots of the 212 walnut samples after pre-processing combination H7 (see Table 4): (**a**) PC 1 vs. PC 4; (**b**) PC 2 vs. PC 4; (**c**) PC 3 vs. PC 4.

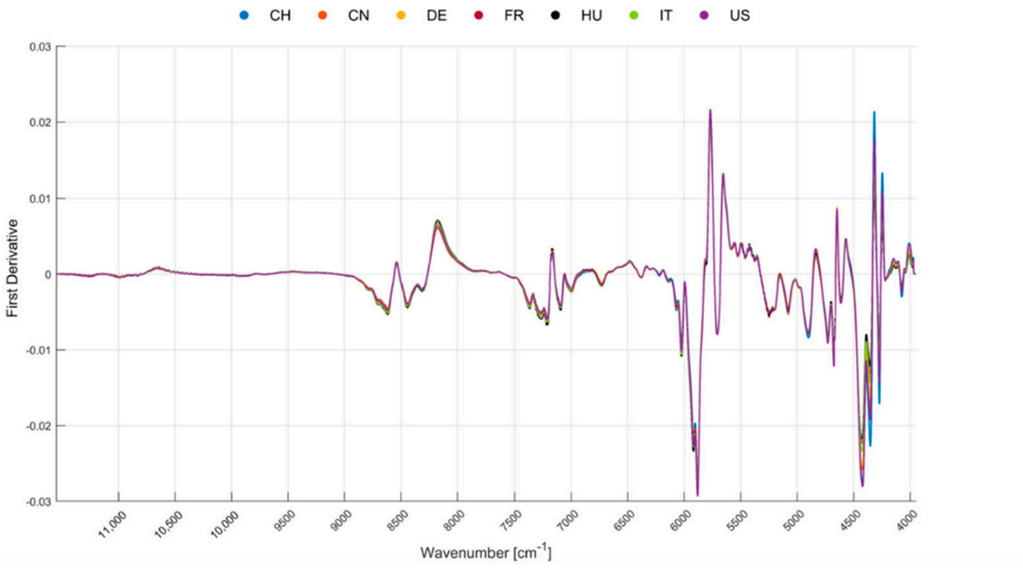

Figure 2. First derivative of MSC-corrected mean spectra of the seven analyzed walnut origins in a wavenumber range of 11,550–3,950 cm^{-1}.
